# Previously undiagnosed hereditary spherocytosis in a patient with jaundice and pyelonephritis: a case report

**DOI:** 10.1186/s13256-016-1144-8

**Published:** 2016-12-01

**Authors:** Yuki Tateno, Ryoji Suzuki, Yukihiro Kitamura

**Affiliations:** Miyake Central Clinic, 1859-16, Kamitsuki, Miyake, Tokyo, Japan

**Keywords:** Hereditary spherocytosis, Jaundice, Anemia, Hemolytic disease

## Abstract

**Background:**

Hereditary spherocytosis is autosomal dominant inherited extravascular hemolytic disorder and is the commonest cause of inherited hemolysis in northern Europe and the United States. The classical clinical features of hereditary spherocytosis are anemia, jaundice, and splenomegaly. However, all of these classical features are not always revealed in the case of mild hemolysis or when hemolysis is well compensated. Patients with hereditary spherocytosis may remain undiagnosed for years if their hemolysis is mild.

**Case presentation:**

A 42-year-old Asian woman presented to our clinic with a sudden onset of high fever with shaking chills and jaundice, suggesting septicemia; however, following detailed investigation, the patient was diagnosed with pyelonephritis and accelerated hemolysis of hereditary spherocytosis due to infection.

**Conclusions:**

It is important to note that transient anemia or jaundice can sometimes be the only initial presenting symptoms in cases of undiagnosed latent hereditary spherocytosis. This case also highlights the fact that physicians should consider concomitant hemolytic disease in patients in whom jaundice and infections that rarely cause jaundice coexist.

## Background

Hereditary spherocytosis (HS) is an autosomal dominant inherited extravascular hemolytic disorder and is the commonest cause of inherited hemolysis in northern Europe and the United States [1]. The classical clinical features of HS are anemia, jaundice, and splenomegaly [1, 2]. However, all of these classical features are not always revealed in the case of mild hemolysis or when hemolysis is well compensated, because the bone marrow is able to increase red cell output. Patients with HS may remain undiagnosed for years if their hemolysis is mild.

## Case presentation

A 42-year-old Asian woman presented to our clinic with a sudden onset of high fever with shaking chills and jaundice. The patient was in her usual good health until 5 days earlier, when she started to have chills and fever. Jaundice had become manifest 2 days earlier. She had a past medical history of cholangitis due to gallbladder stones, for which she had undergone laparoscopic cholecystectomy 3 months before. She had no family history of hemolytic diseases. She was alert; her temperature was 39.3 °C, her blood pressure was 122/61 mmHg, and her pulse rate was regular at 119 per minute. Her respiration rate was within normal range. Her physical examination revealed icteric skin and sclera, but she had no abdominal or costovertebral angle tenderness. There were no other specific findings in her physical examination. A routine hematological examination showed normocytic normochromic anemia with hemoglobin 9.0 g/dl. The patient’s reticulocyte count was elevated at 25.0%, her white blood cell count was 10,580/μl, and her blood platelet count was 125,000/μl. The patient’s liver function test values were elevated: total bilirubin 10.9 mg/dl with direct bilirubin of 6.0 mg/dl, aspartate aminotransferase 49 IU/L, alanine aminotransferase 65 IU/L, alkaline phosphatase 441 IU/L, lactate dehydrogenase 550 IU/L, and γ-glutamyl transferase 301 IU/L. Other blood chemistry findings were all within normal limits. Coagulation test results were within normal limits, with a prothrombin time 89% of normal and an activated partial thromboplastin time of 26.0 seconds. A blood culture was positive for *Escherichia coli*.

The initial clinical impression was septicemia due to acute obstructive suppurative cholangitis caused by a combination of high fever, jaundice, and elevated liver function values accompanied with hyperbilirubinemia in which direct bilirubin was dominant. The patient immediately underwent abdominal contrast-enhanced computed tomography, which revealed splenomegaly and a distended left kidney. However, no signs of biliary tract obstruction were present. At this point, urinalysis was performed to evaluate the possibility of pyelonephritis. The urinalysis results were 3+ test for leukocytes, 2+ for nitrate, 3+ for hemoglobin, 2+ for bilirubin, and 3+ for urobilinogen. The patient’s urinary sediment was loaded with white cells and bacteria and contained five to nine erythrocytes per high-power field. Her urine culture was positive for *E. coli*. The urinalysis findings suggested the diagnosis of septicemia due to pyelonephritis; however, the cause of the patient’s hyperbilirubinemia was unclear. Other infections accompanied with hyperbilirubinemia, such as infectious mononucleosis, malaria, and Weil syndrome (leptospirosis), were unlikely in light of negative findings of the microbiological examination and peripheral blood smear.

**Fig. 1:**
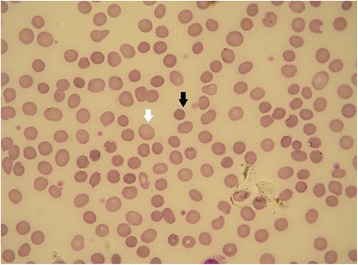
The patient’s peripheral blood smear on admission. The *black arrow* shows a spherocyte. The *white arrow* shows a normal red blood cell

The patient was supposed to have concurrent hemolysis because of anemia, hyperbilirubinemia, and urobilinogenuria. To substantiate the diagnosis of hemolysis, the following laboratory examinations were performed. The patient’s haptoglobin level was 8 mg/dl (normal range 19–200 mg/dl), and the results of direct and indirect Coombs tests were negative. A peripheral blood smear showed normal red blood cells with spherocytosis (Fig. 1).

On the basis of the peripheral blood smear findings, splenomegaly and the patient’s past history of cholelithiasis, HS was strongly suspected. An osmotic fragility test was performed, which showed increased fragility. Thus, the diagnosis of undiagnosed HS was confirmed, and the patient’s clinical condition was diagnosed as pyelonephritis and accelerated hemolysis of HS due to infection. Her family members were queried whether they had HS, according to the patient’s wish, and her father was diagnosed with HS on the basis of a peripheral blood smear and an osmotic fragility test. The patient was treated with meropenem as an intravenous antibiotic for 14 days and with oral cephalexin for 30 days. She recovered well and was discharged 18 days after admission. Fourteen days after discharge, her laboratory values normalized. It was decided that if she experienced chronic severe hemolysis and anemia, a splenectomy would be scheduled.

## Discussion

To our knowledge, this is the first description of a case of significant jaundice due to excessive hemolysis induced by pyelonephritis based on undiagnosed HS. The patient presented with hemolysis and jaundice in the presence of pyelonephritis. Our initial clinical impression was septicemia due to acute obstructive suppurative cholangitis caused by a combination of high fever, jaundice, and elevated liver function values accompanied with hyperbilirubinemia. If HS had not been recognized, unnecessary and invasive procedures such as endoscopic retrograde cholangiopancreatography or emergency operation might have been done.

To substantiate a diagnosis of HS, several laboratory examinations were performed. Autoimmune hemolytic anemia can also cause hemolysis; however, it was excluded because the results of direct and indirect Coombs tests were negative. Thrombotic thrombocytopenic purpura (TTP) and hemolytic uremic syndrome (HUS) should be considered in the differential diagnosis when encountering the combination of hemolysis, slight thrombocytopenia, and jaundice in the presence of pyelonephritis. However, the absence of neurological symptoms, kidney failure, and abnormal red blood cells such as burr cells or helmet cells excluded TTP and HUS in our patient. Ultimately, on the basis of the patient’s spherocytosis, splenomegaly, and past history of cholelithiasis, HS was strongly suspected.

HS is an autosomal dominant inherited extravascular hemolytic disorder and is the commonest cause of inherited hemolysis in northern Europe and the United States [1]. The pathological cause of HS is deficiency or dysfunction of proteins of the red cell membrane. The molecular defect may involve the genes encoding spectrin, ankyrin, band 3, or protein 4.2. Deficiency or dysfunction of any of these proteins, all of which are involved in the attachment of the cytoskeleton to the membrane integral domain, results in a loss of surface area and leads to spheroidal, osmotically fragile red blood cells that are selectively trapped in the spleen and destroyed [1]. HS itself is not a life-threatening disease; however, two comorbidities can induce a serious medical condition in patients with HS.

The most alarming comorbid disease in patients with HS is parvovirus B19 infection. In patients with HS, parvovirus B19 infection leads to severe red cell aplasia and a sudden, profound fall in hemoglobin level, and transfusion is sometimes required [1]. Patients with HS who have not had parvovirus infection should be warned of possible future infection.

The second is cholelithiasis, which is prone to coexist with HS. Hemolysis is associated with increased red cell turnover and an increased pigment load for the liver, and this may result in the development of gallstones [1]. Gallbladder stones can cause cholecystitis, cholangitis, or pancreatitis, and these diseases sometimes require operation or therapeutic endoscopy.

The classical clinical features of HS are anemia, jaundice, and splenomegaly [1, 2]. However, all of these classical features are not always revealed in the case of mild hemolysis or when hemolysis is well compensated, because the bone marrow is able to increase red cell output [1, 2]. Actually, patients with HS may remain undiagnosed for years if their hemolysis is mild.

An increase in hemolysis, which presents as transient anemia or jaundice, is sometimes produced by stress or other infections [1, 2]. Activation of the reticuloendothelial system during infection is thought to bring about this increase [3]. The transient anemia or jaundice can be the only initial presentation for patients with undiagnosed latent HS. If HS is clinically suspected, the peripheral blood smear is the simplest but most important examination [2]. Routine hematological and blood chemical tests such as hemoglobin, reticulocyte, bilirubin, and direct and indirect Coombs tests are also helpful [2]. An osmotic fragility test is sometimes performed to make a definitive diagnosis; however, it is time-consuming and labor-intensive, and it is not essential for making the diagnosis if there are obvious spherocytes [1, 2]. The process of diagnosis is not difficult if physicians do not miss the clinical signs of HS and suspect it. In other words, to reveal undiagnosed HS, awareness of HS is most important in cases of jaundice and/or anemia accompanied with infections.

## Conclusions

Especially when jaundice is present with infections that rarely cause jaundice by themselves, physicians should consider the concomitance of hemolytic disease. In addition, the existence of splenomegaly and a past history of cholelithiasis should raise suspicion for HS.
